# Anatomical and functional outcomes of subthreshold micropulse laser versus intravitreal ranibizumab injection in treatment of diabetic macular edema

**DOI:** 10.1186/s40942-020-00265-6

**Published:** 2020-12-03

**Authors:** Amr Abdelrahman, Wagiha Massoud, Ahmed Mohamed Kamal Elshafei, Mahmoud Genidy, Raafat Mohyeldeen Abdelrahman Abdallah

**Affiliations:** Ophthalmology Department, Faculty of Medicine, Minia University, Minia, Egypt

**Keywords:** Diabetic macular edema, Multifocal electroretinogram, Subthreshold micropulse laser, Ranibizumab

## Abstract

**Background:**

To compare the therapeutic effects of subthreshold micropulse laser (SML) versus intravitreal injection of ranibizumab in treatment of diabetic macular edema (DME) both anatomically using optical coherence tomography (OCT) and functionally using best corrected visual acuity (BCVA) and multifocal electroretinogram (mfERG).

**Methods:**

his study was an interventional prospective randomized comparative study. The study included 120 eyes classified into 3 groups: Group 1 included 40 eyes of 28 patients treated by SML laser, group 2 included 40 eyes of 32 patients treated by intravitreal injection of ranibizumab, and group 3 (control group for mfERG) included 40 eyes of 20 patients with diabetes mellitus (DM) of more than 10 year duration with no signs of diabetic retinopathy (DR). BCVA measurements, OCT and mfERG were done for the cases before and after interference and were followed up for 6 months

**Results:**

By the end of the follow up period, BCVA significantly improved by 31% in group 1 vs 93% in group 2 with a statistically highly significant difference between the two groups (*p *value < 0.001). There was also a significant decrease in central subfield thickness in both groups with more reduction in group 2 compared with group 1 (*p *value < 0.001). There was a significant improvement in P1 amplitude of mf-ERG in group 2 (*p *value < 0.002) with no significant improvement in group 1. There was a significant decrease in P1 implicit time in group 2 (*p *value < 0.001) while there was no significant decrease in group1.

**Conclusions:**

Intravitreal injection of ranibizumab is a superior treatment modality for DME compared with SML regarding both anatomical and functional outcomes.

*Trial registration: *This study has been approved by the local ethical committee of faculty of medicine of Minia University and retrospectively registered at the clinical trial gov. with Identifier: NCT04332133.

## Introduction

Diabetic macular edema (DME) causes significant visual loss in diabetic patients. About 20% and 40% of patients with type 1 and type 2 diabetes mellitus (DM), respectively, develop DME. One-third of diabetic patients who have had DM for more than twenty years will develop DME [1]. Early impairment in the function of the middle and inner layers of the retina has been reported in diabetic patients before appearance of vascular complications [2]. A good independent guide of macular function in patients with DME is multifocal electroretinogram (mfERG) readings from the macular area, which strongly associate with morphologic alterations in the macula [3]. Some investigators suggested that temporal characteristic (implicit time) of mfERG waves are more important than amplitudes for evaluation of retinal function in diabetic patients. They concluded that patients with DM show temporal changes indicating delayed neural transmission due to local impairment of blood glucose metabolism [4, 5]. In contrast, others emphasize the importance of both parameters (implicit time and amplitude) in identifying retinal affection in DM [6, 7].

Intravitreal (IV) injections of anti-vascular endothelial growth factor (VEGF) agents provide good visual outcomes in treatment of DME. However, IV anti-VEGF injections are expensive, need to be repeated many times and have the potential risk of causing endophthalmitis [8]. Subthreshold micropulse laser (SML) treatment of DME has the same effect as conventional laser treatment, nonetheless, there is less damage to adjacent tissues of the burn area in the retinal pigment epithelium (RPE) [9]. SML allows laser emission to be divided into bursts of short cyclic pulses that remain for microseconds permitting substantial cooling among these short pulses [10]. The aim of this study is to compare between the anatomical and functional results of IV injection of ranibizumab and SML in treatment of DME both anatomically by spectral domain optical coherence tomography (SD-OCT) and functionally by best-corrected visual acuity (BCVA) and mfERG.

## Patients and methods

This study was an interventional prospective randomized comparative study performed in the Ophthalmology Department, Minia University Hospital, Minia-Egypt, between December 2016 and March 2019. The study was approved by the local ethical committee of Minia University and was adherent to the tents of Declaration of Helsinki. After explanation of the aim and methodology of the study and its possible risks and benefits, an informed consent was obtained from each patient. The study included 80 eyes of 60 patients with DME (28 males and 32 females) and 40 eyes of 20 diabetic patients (≥ 10 years duration) without diabetic retinopathy (10 males and 10 females) as a control group for mfERG measurements. The included patients had DME defined as the presence of intraretinal and / or subretinal fluid involving the fovea on OCT with BCVA < 0.5 decimal Snellen acuity with controlled blood glucose level confirmed by glycosylated hemoglobin (HbA_1c_) < 6.5%. Exclusion criteria included patients with history of previous intraocular surgery, laser treatment, IV injection, macular disease or ischemia, proliferative diabetic retinopathy, vitreoretinal traction, interruption of external limiting membrane (ELM) or ellipsoid zone (EZ) on SD OCT, dense media opacity, optic disc pathology or other ocular pathology and those with history of strokes or ischaemic heart diseases. Also, patients with central subfield thickness (CST) > 400 µm on OCT were excluded from the study.

All patients were subjected to complete history taking including; duration of DM, past glycemic control (HbA_1_c), medications, general medical history (e.g., renal disease, systemic hypertension, serum lipid levels and pregnancy), history of trauma, other eye diseases, IV injections, laser photocoagulation and ocular surgery. Full ophthalmological examination was performed. Fluorescein angiography was done at baseline for all patients using TOPCON TRC-XXX fundus camera (Topcon Corporation, IMAGE net 200, Tokyo, Japan) to detect macular leakage and ischemia. OCT examination was done using the Cirrus HD-OCT 4000 platform (Carl Zeiss Meditec AG, Jena, Germany). Both CST which represents the thickness of the central 1 mm diameter circular zone indicating the foveal area (normal value is up to 220 microns) and macular cube average volume were measured before and after treatment of DME by IV injection of ranibizumab or SML. Recording of mf-ERG was done using the RetiPort/Scan 2 System (Roland Consult, Brandenburg, Germany) according to the International Society for Clinical Electrophysiology of Vision (ISCEV) standards. [11] BCVA was measured using Snellen chart then converted to decimal acuity for statistical analysis.

The treated eyes were randomly classified into two groups. Randomization was performed using computer generated tables. Group 1 included 40 eyes of 28 patients that were treated by SML. Laser treatment sessions were done using IRIDEX IQTM 532 nm (Mountain View, CA, USA). Mainster focal grid contact lens (× 1.05 magnification) was applied. Fixed treatment parameters were used in all cases: 200 ms exposure duration, 200 µm spot size, 400 mW powers, and a 5% duty cycle. Duty cycle refers to the on and off cycles of micropulse technology. Laser was applied 10 ms “on” and 190 ms “off”. The laser was applied using a 7 × 7 grid pattern with zero spacing and the entire area between the arcade was treated including the fovea. Follow up of this group (1) was performed 6 months after SML treatment. Group (2) included 40 eyes of 32 patients (24 unilateral and 8 bilateral) that received IV ranibizumab according to the pro re nata (PRN) protocol. All patients received 3 monthly injections and then followed monthly for a total of 6 months from the initial treatment visit. Retreatment was performed if the fluid persisted or worsened compared to baseline on OCT and/or BCVA less than 0.5 in presence of residual macular edema (26 eyes). The follow up evaluation included BCVA measurement and OCT imaging. Recording of implicit time and amplitude of P1of mfERG was done at baseline and after 6 months in both treated groups. Group 3 (the control group) was subjected to complete ophthalmological evaluation, OCT examination and mf ERG. mfERG amplitudes and implicit time of P1 were compared between control subjects and the patients of group1 and 2.

### Statistical analysis

Statistical analysis was done using IBM SPSS Statistics for Windows, Version 25.0 (IBM Corp., Armonk, NY, USA). One-way ANOVA test was used for parametric quantitative data between the 3 groups. Independent sample *t* test was used for parametric quantitative data between 2 groups. Paired sample t test was used for parametric quantitative data within each group. *P* value was significant if < 0.05.

## Results

Group 1 included 40 eyes of 28 patients (16 unilateral and 12 bilateral). The patients were 15 females and 13 males with mean age of 61.7 ± 1.7 years and mean duration of DM of 12.5 ± 3.2 years. Group 2 included 40 eyes of 32 patients (24 unilateral and 8 bilateral). The patients were 17 females and 15 males with mean age was 61.5 ± 3.3 years and the mean duration of DM was 13.9 ± 3.8 years. The control group included 40 eyes of 20 diabetic patients (10 males and 10 females) without diabetic retinopathy with mean age of 62.4 ± 5.2 years and mean duration of DM of 12 ± 4.2 years. There was no significant difference concerning sex, age or DM duration between the 3 groups. In group 1, BCVA significantly improved from 0.43 ± 0.01 at base line to 0.56 ± 0.16 (*p* < 0.001) and the percentage of improvement was 31 ± 26.1%. On the other hand, in group 2, BCVA significantly improved from 0.14 ± 0.07 at base line to 0.26 ± 0.09 (*p* < 0.001) and the percentage of improvement was 97.6 ± 46.6%. There was a significant difference in the percentage of improvement in BCVA between the two groups (*p* < 0.001).

As regard OCT, in Group 1, CST significantly decreased from 338.7 ± 11.8 µm at baseline to 299.1 ± 12.9 µm at 6 months (*p* < 0.001) and the percentage of decrease was 11.69%. On the other hand, in Group 2, CST decreased from 359.8 ± 26.7 µm at baseline to 235.1 ± 52.4 µm at 6 months (*p* < 0.001) and the percent of reduction was 34.66%. Also, OCT cube average volume significantly decreased in group 1 from 366.7 ± 16.4 µm at base line to 329.6 ± 20.8 µm at 6 months (*p* < 0.001) and the percentage of reduction was 10.11%. In Group 2, cube average volume significantly decreased from 345.8 ± 45.4 µm at base line to 249.7 ± 34.5 µm at 6 months (*p* < 0.001) and the percentage of reduction was 27.7%. There was a statistically highly significant difference in reduction of both OCT parameters between the 2 groups with more reduction in group 2 (p < 0.001). The average number of injections for group 2 was 4.5 ± 1.13.

As regard mfERG, the mean P1 amplitude of the central ring before treatment in group 1 was 36.6 ± 5.2 nv/deg^2^ (range: 29.2–54.3) and in group 2 it was 34.6 ± 9.7 nv/deg^2^ (range: 24.3–53.9) compared to 64.6 ± 7 nv/deg^2^ (range: 49.1–75.8) in the control group with a highly significant difference (p < 0.001).

By the end of the 6th month after treatment, in group1 there was a minimal increase of the mean P1 amplitude of the central ring to 37.9 ± 5 nv/deg^2^ (range: 24.7–49.3). This increase was not significant (*p* = 0.122). On the other hand, the mean P1 amplitude of central ring in group 2 increased to 40.5 ± 10.1 nv/deg^2^ (range: 27.4–63.4) with a significant difference between P1 amplitude before and after treatment (*p* < 0.001). The mean percentage of increase of P1 amplitude was 3.55% in group 1 compared to 17.1% in group 2 with a significant difference between the two groups (*p* value < 0.002).

**Fig. 1:**
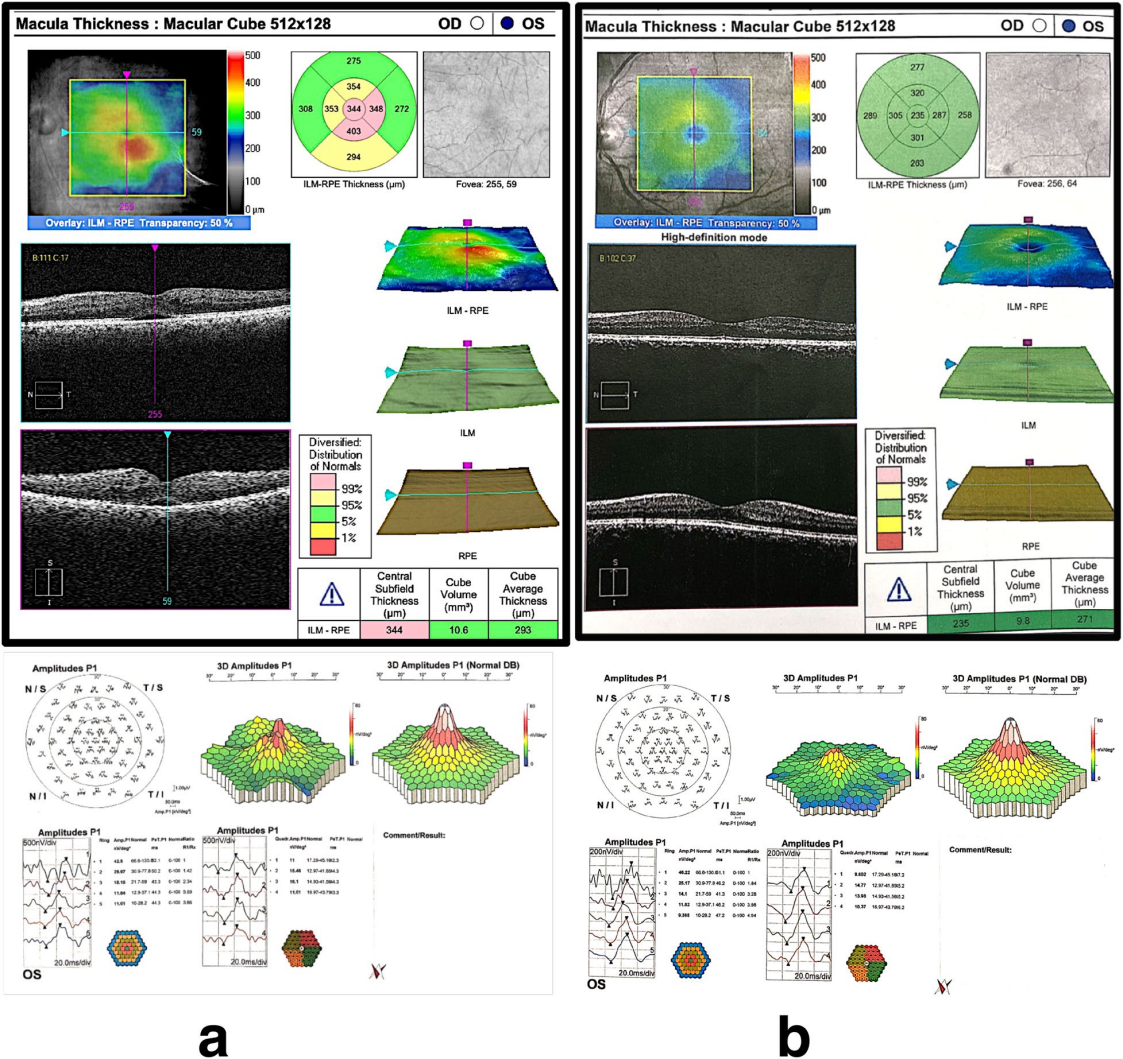
OCT and mfERG **a** Images of a patient from group1 with baseline OCT revealed diffuse retinal thickness, neurosensory detachment, central subfield thickness of 344um and cube average thickness of 293 um. The baseline p1 amplitude of mfERG was 42.50nv/deg^2^. **b** The images of the same patient 6 months after treatment by SML with a reduction of OCT central subfield thickness to 235 um and cube average thickness to 271 um while the p1 amplitude of mfERG increased to 46.22 nv/deg^2^

**Fig. 2:**
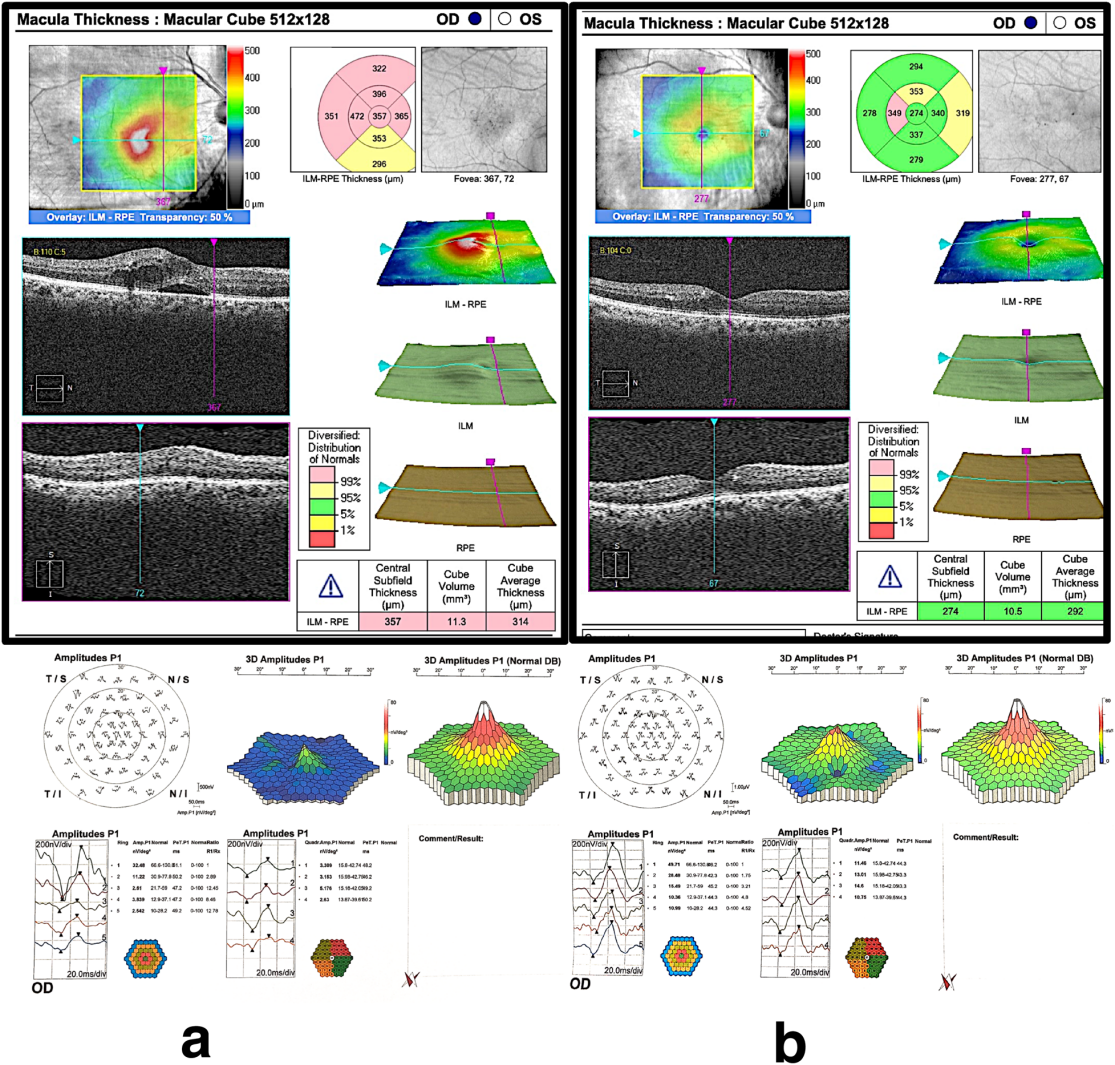
OCT and mfERG **a** Images of a patient from group 2 with baseline OCT revealed cystoid macular edema with neurosnsory detachment, central subfield thickness of 357 um and Cube average thickness 314um. The baseline mfERG p1 amplitude was 32.48 nv/deg^2^. **b** The same patient 6 months after treatment with IV injections of ranibizumab. OCT revealed resolution of the cystoid macular edema and neurosnsory detachment with central subfield thickness reduced to 274 um and cube average thickness reduced to 292 um. The p1 amplitude of mfERG improved to 49.71 nv/deg^2^

The mean P1implicit time of the central ring of mfERG was minimally decreased in group 1, from 47.8 ± 1.6 ms (range: 46.1–50.3) to 47.1 ± 3.1 ms (range: 42.3–54.3). This decrease was statistically insignificant (*p *= 0.333). In group 2 it decreased from 48.7 ± 23 ms (range: 42.53–59.6) before treatment to 46.8 ± 2.5 ms (range: 41.6–51.2) at 6 months after treatment. This decrease was highly significant (*p* < 0.001). The mean percentage of decrease of P1 implicit time was 1.4% in group 1 compared to in 3.9% in group 2 with statistically insignificant difference between the two groups (*p* = 0.11). There was a significant strong positive correlation between BCVA and P1 amplitude both at baseline and at the end of treatment *(r=*0.77 and 0.71, respectively). On the other hand, there was a significant negative moderate correlation between CST and P1 amplitude at base line and at the end of treatment (*r=*− 0.66 and − 0.64 respectively). Figure 1a and b represents an example of OCT scan, CST and mf-ERG at baseline and 6 months after SML while Fig. 2a and b represents a similar example of OCT scan, CST and mf-ERG at baseline and 6 months after IV ranibizumab injection.

## Discussion

Laser photocoagulation was considered the standard treatment of DME for many years. However, variable degrees of complications are encountered with this treatment modality. Currently, intravitreal injection of anti VEGF with or without laser photocoagulation is the standard line of management for patients with DME [12]. Newer methods of laser such as SML are under investigation to improve the efficacy while reducing the adverse effects [13].

In the current study, intravitreal injection of ranibizumab was compared with macular SML for treatment of DME both structurally using OCT and functionally using BCVA and mfERG.

Patients with CST more than 400um were excluded from the study as it is well known that SML is less effective in such cases [14, 15].

Structurally, there was a highly significant reduction in CST and macular cube average volume in both groups by the end of the follow-up period, with significantly more reduction in both parameters among the IV injection group.

Functionally, there was a significant improvement of BCVA by the end of the follow up period compared with the baseline data in both groups. Also, more improvement was achieved in the ranibizumab group compared with SML group with a highly significant difference between both groups.

The results of ranibizumab injection were in accordance with previous studies that evaluated anatomical and functional effects of treatment of DME by intravitreal injection of ranibizumab according to changes in BCVA and such as RESOLVE, RESTORE, RIDE and RISE and Chun et al. studies [15–18]. Results in of SML were in accordance with many previous studies that used SML for treatment of DME with CST improvement during follow-up [19–22].

However, anatomical improvement is not always associated with improvement in functional outcomes. OCT can measure the degree of edema, but the cellular damage cannot be evaluated. According to Browning et al., several eyes with DME had good vision while many eyes with normal CST had decreased vision [23]. OCT, however, can provide some biomarkers that give an indication about the functional outcomes.

In this study, the functional evaluation was done both subjectively using BCVA and objectively by mfERG. Yamamoto et al. showed that mf-ERG readings from the macular area were good objective indicators of macular function in patients with DME and were strongly correlated with the morphologic changes in the macula [3]. On the other hand, Dale et al. revealed that there was a considerable disagreement between OCT and mfERG as the latter tends to miss small local abnormalities that are detectable on OCT. Conversely, OCT can appear normal with clearly abnormal mfERG results. In some cases, functional damage may appear in mfERG before structural changes could be detected with OCT [24].

Regarding mfERG, with IV injection of ranibizumab, there was 17.1% increase in the P1 amplitude by the end of the follow-up period and this increase was highly significant. A statistically significant decrease was noted in the P1 implicit time of central ring compared with the baseline and the percentage of decrease was 3.9%. This significant improvement in both electrophysiological parameters of P1 could be explained by the concept that the significant decrease in the macular edema results in enhancement of synaptic connectivity indicating that intravitreal injection of ranibizumab enhances inner retinal function recovery with the reduction of macular edema [23].

These results were in accordance with YuDong et al. who showed a significant increase of mean amplitude of P1 in the central ring at all examinations compared with the baseline. The mean P1 implicit time in the central ring was shortened, but not significantly . On the other hand, the results of the current study were not in agreement with Barbara et al. who studied 17 eyes of 17 patients with type 2 DM and DME that were treated with intravitreal injections of ranibizumab. The mean P1-response density in R1 increased, however, the increase was statistically insignificant, and the mean P1-implicit time also did not differ significantly in comparison with the baseline [26]. This difference in mf-ERG responses could be explained according to Baget et al. [27], who found that eyes with cystoid and spongiform DME had a better response density compared to the serous type at baseline. Similarly, eyes with high inner segment/outer segment (IS/OS) and external limiting membrane (ELM) preservation rates presented greater early response density in relation to the others [27]. In the current study, patients with vitreoretinal traction, interruption of ELM or IS/OS junction and cases of proliferative DR were excluded from the study to avoid selection bias that would affect treatment results among both groups and this could explain significant improvement in mfERG. Therefore, the use of OCT for DME evaluation before testing mfERG may give more reliable results.

In SML group the significant improvement of BCVA and reduction of CST were in accordance with Pei-Pei et al. [13], who compared BCVA and CST after 532-nm subthreshold and threshold laser grid photocoagulation for the treatment of DME. In subthreshold group, CST significantly declined from 364um at baseline to 340um after 3 months and 320um after 6 months.

In this study, after SML treatment both P1 amplitude and implicit time showed no significant changes between baseline and 6 months after treatment. This was in accordance with Venkatesh et al., who compared the efficacy of SML with double-frequency neodymium YAG laser in management of clinically significant DME. They noted insignificant change in P1 amplitude after SML treatment [28].

In the current study, there was a significant decrease in P1 amplitude and significant increase in implicit time between both treated groups (diabetic patients with DME) and the control group (diabetic patients with no diabetic retinopathy) indicating that DME affects both electrophysiological parameters of mfERG. These results agree with earlier studies that reported abnormal mfERG parameters in patients with DME [29–31].

Many randomized clinical trials proved the effectiveness of intravitreal injection of ranibizumab for treatment of DME. However, it has an economic burden and potential risks. In this study, intravitreal injection was compared with another treatment modality which is SML. The later has several advantages over conventional laser photocoagulation as it is invisible retinal phototherapy with no retinal damage by laser and consequently there is no inflammatory response or loss of retinal function. It is well tolerated by the patients with no or minimal pain sensation during the laser procedure.

A major disadvantage of SML treatment is lack of reliable titration protocols to achieve subvisible treatments. However, SML tissue-sparing therapy may play a major role in the management of DME in the future, especially when considering combining it with intravitreal injections. This regimen may be helpful in reducing the number of injections needed to control DME. Limitations of this study include the relatively short follow up time and small sample size. Studies with longer follow up period are needed to verify the long-term results of both procedures.

## Conclusions

Intravitreal injection of ranibizumab is a more superior treatment modality compared with SML concerning the improvement of both the anatomical and functional results in patients with diabetic DME.

## Data Availability

The datasets used/or analysed during the current study are available from the corresponding author on reasonable request.

## Abbreviations

SML: Subthreshold micropulse laser
DME: Diabetic macular edema
OCT: Optical coherence tomography
BCVA: Best corrected visual acuity
mfERG: Multifocal electroretinogram
DM: Diabetes mellitus
IV: Intravitreal
VEGF: Vascular endothelial growth factor
SD OCT: Spectral domain optical coherence tomography
HbA1c: Glycosylated hemoglobin
RPE: Retinal pigment epithelium
CST: Central subfield thickness
ELM: External limiting membrane
EZ: Ellipsoid zone

## References

[1] Klein R, Knudtson MD, Lee KE, Gangnon R, Klein BE (2009). The Wisconsin epidemiologic study of diabetic retinopathy XXIII: The twenty-five-year incidence of macular edema in persons with type 1 diabetes. Ophthalmology.

[2] Lung JC, Swann PG, Wong DS, Chan HH (2012). Global flash multifocal electroretinogram: Early detection of local functional changes and its correlations with optical coherence tomography and visual field tests in diabetic eyes. Doc Ophthalmol.

[3] Yamamoto S, Yamamoto T, Hayashi M, Takeuchi S (2001). Morphological and functional analyses of diabetic macular edema by optical coherence tomography and multifocal electroretinograms. Graefes Arch Clin Exp Ophthalmol.

[4] Fortune B, Schneck ME, Adams AJ (1999). Multifocal electroretinogram delays reveal local retinal dysfunction in early diabetic retinopathy. Invest Ophthalmol Vis Sci.

[5] Han Y, Bearse MA, Schneck ME, Barez S, Jacobsen CH, Adams AJ (2004). Multifocal electroretinogram delays predict sites of subsequent diabetic retinopathy. Invest Ophthalmol Vis Sci.

[6] Greenstein VC, Holopigian K, Hood DC, Seiple W, Carr RE (2000). The nature and extent of retinal dysfunction associated with diabetic macular edema. Invest Ophthalmol Vis Sci.

[7] Harrison WW, Bearse MA, Schneck ME, Wolff BE, Jewell NP, Barez S (2011). Prediction, by retinal location, of the onset of diabetic edema in patients with nonproliferative diabetic retinopathy. Invest Ophthalmol Vis Sci.

[8] Rosenfeld PJ, Flynn HW, Schwartz SG (2011). Endophthalmitis after intravitreal injection of vascular endothelial growth factor antagonists. Six-year experience at a university referral center. Retina.

[9] Ohkoshi K, Yamaguchi T (2010). Subthreshold micropulse diode laser photocoagulation for diabetic macular edema in Japanese patients. Am J Ophthalmol.

[10] Reyes-Torres Pedro, Reyes-Torres Javier, Baget-Bernaldiz Marc, Blasco-Suñe Cristina (2014). Laser Treatment for Diabetic Macular Edema in the 21st Century. Curr Diabetes Rev.

[11] McCulloch DL, Marmor MF, Brigell MG, Hamilton R, Holder GE, Tzekov R, Bach M (2015). ISCEV Standard for full-field clinical electroretinography (2015 update). Documenta ophthalmologica Advances in ophthalmology.

[12] Vujosevic S, Berton M, Bini S, Casciano M, Cavarzeran F, Midena E (2016). Hyperreflective retinal spots and visual function after anti-vascular endothelial growth factor treatment in center-involving diabetic macular edema. Retina.

[13] Pei-Pei W, Shi-Zhou H, Zhen T, Lin L, Ying L, Jiexiong O (2015). Randomised clinical trial evaluating best-corrected visual acuity and central macular thickness after 532-nm subthreshold laser grid photocoagulation treatment in diabetic macular oedema. Eye.

[14] Diabetic Retinopathy Clinical Research Network (2010). Randomized trial evaluating ranibizumab plus prompt or deferred laser or triamcinolone plus prompt laser for diabetic macular edema. Ophthalmology.

[15] Mitchell P, Bandello F, Schmidt-Erfurth U, Lang GE, Massin P, Schlingemann RO (2011). The RESTORE study: Ranibizumab monotherapy or combined with laser versus laser monotherapy for diabetic macular edema. Ophthalmology.

[16] Massin P, Bandello F, Garweg JG (2010). Safety and efficacy of ranibizumab in diabetic macular edema (RESOLVE study): a 12-month, randomized, controlled, double-masked, multicenter phase II study. Diabetes Care.

[17] Nguyen QD, Brown DM, Marcus DM, Boyer DS, Patel S, Feiner L (2012). Ranibizumab for diabetic macular edema: Results from 2 phase III randomized trials: RISE and RIDE. Ophthalmology.

[18] Chun DW, Heier JS, Topping TM, Duker JS, Bankert JM (2006). A pilot study of multiple intravitreal injections of ranibizumab in patients with center-involving clinically significant diabetic macular edema. Ophthalmology.

[19] Friberg TR, Karatza EC (1997). The treatment of macular disease using a micropulsed and continuous wave 810-nm diode laser. Ophthalmology.

[20] Laursen ML, Moeller F, Sander B, Sjoelie AK (2004). Subthreshold micropulse diode laser treatment in diabetic macular oedema. Br J Ophthalmol.

[21] Luttrull JK, Musch DC, Mainster MA (2005). Subthreshold diode micropulse photocoagulation for the treatment of clinically significant diabetic macular oedema. Br J Ophthalmol.

[22] Luttrull JK, Sinclair SH (2014). Safety of subthreshold diode micropulse laser for fovea- involving diabetic macular edema in eyes with good visual acuity. Retina.

[23] Browning J, Glassman R, Aiello P, Beck W, Brown M, Fong S, Bressler M, Danis P, Kinyoun L, Nguyen D (2007). Diabetic Retinopathy Clinical Research Network, relationship between optical coherence tomography-measured central retinal thickness and visual acuity in diabetic macular edema. Ophthalmology.

[24] Dale EA, Hood DC, Greenstein VC, Odel JG (2010). A comparison of multifocal ERG and frequency domain OCT changes in patients with abnormalities of the retina. Doc Ophthalmol.

[25] Fu YD, Wang P, Du XX, Wang DB (2017). Structural and functional assessment after intravitreal injection diabetic macular edema. Doc Ophthalmol.

[26] Nowacka -Barbara, Kirkiewicz M, Mozolewska K, Piotrowska Wojciech (2016). The macular function and structure in patients with diabetic macular edema before and after ranibizumab treatment. Doc Ophthalmol.

[27] Romero-Aroca -Baget-Marc,P, Bautista-Perez A, Mercado J (2017). Multifocal electroretinography changes at the 1-yearfollowup in a cohort of diabetic macular edema patients treated with ranibizumab. Doc Ophthalmol.

[28] Venkatesh P, Ramanjulu R, Azad R, Vohra R, Garg S (2011). Subthreshold micropulse diode laser and double frequency neodymium: YAG laser in treatment of diabetic macular edema: a prospective, randomized study using multifocal electroretinography. PhotomedLaser Surg.

[29] Tehrani NM, Riazi-Esfahani H, Jafarzadehpur E, Mirzajani A, Talebi H, Amini A, Mazloumi M, Roohipoor R (2015). Riazi- Esfahani M. Multifocal electroretinogram in diabetic macular edema; Correlation with visual acuity and optical coherence tomography. J Ophthalmic Vis Res.

[30] Comyn O, Sivaprasad S, Peto T, Neveu MM, Holder GE, Xing W (2014). A randomized trial to assess functional and structural effects of ranibizumab versus laser in diabetic macular edema (the LUCIDATE study). Am J Ophthalmol.

[31] Weiner A, Christopoulos VA, Gussler CH, Adams DH, Kaufman SR, Kohn HD (1997). Foveal cone function in nonproliferative diabetic retinopathy and macular edema. Invest Ophthalmol Vis Sci.

